# P-1724. Cryptococcus neoformans var. grubii infection due to medical cannabis consumption

**DOI:** 10.1093/ofid/ofaf695.1895

**Published:** 2026-01-11

**Authors:** Kailey L Hughes, Christopher C Marino, Shaoji Cheng, Eileen Driscoll, Kaitlin Phillips, Cornelius J Clancy, M Hong Nguyen

**Affiliations:** University of Pittsburgh, Pittsburgh, PA; UPMC, Pittsburgh, Pennsylvania; University of Pittsburgh, Pittsburgh, PA; University of Pittsburgh, Pittsburgh, PA; UPMC, Pittsburgh, Pennsylvania; University of Pittsburgh, Pittsburgh, PA; University of Pittsburgh Medical Center, Pittsburgh, Pennsylvania

## Abstract

**Background:**

Backgound. Cannabis can be contaminated with fungi and bacteria. Cannabis use is associated with invasive fungal infections (IFIs; aspergillosis in particular) in case reports and epidemioloic studies. However, precise sources of these IFIs, whether directly from cannabis or other environmental foci, have not been validated.

**Figure:**
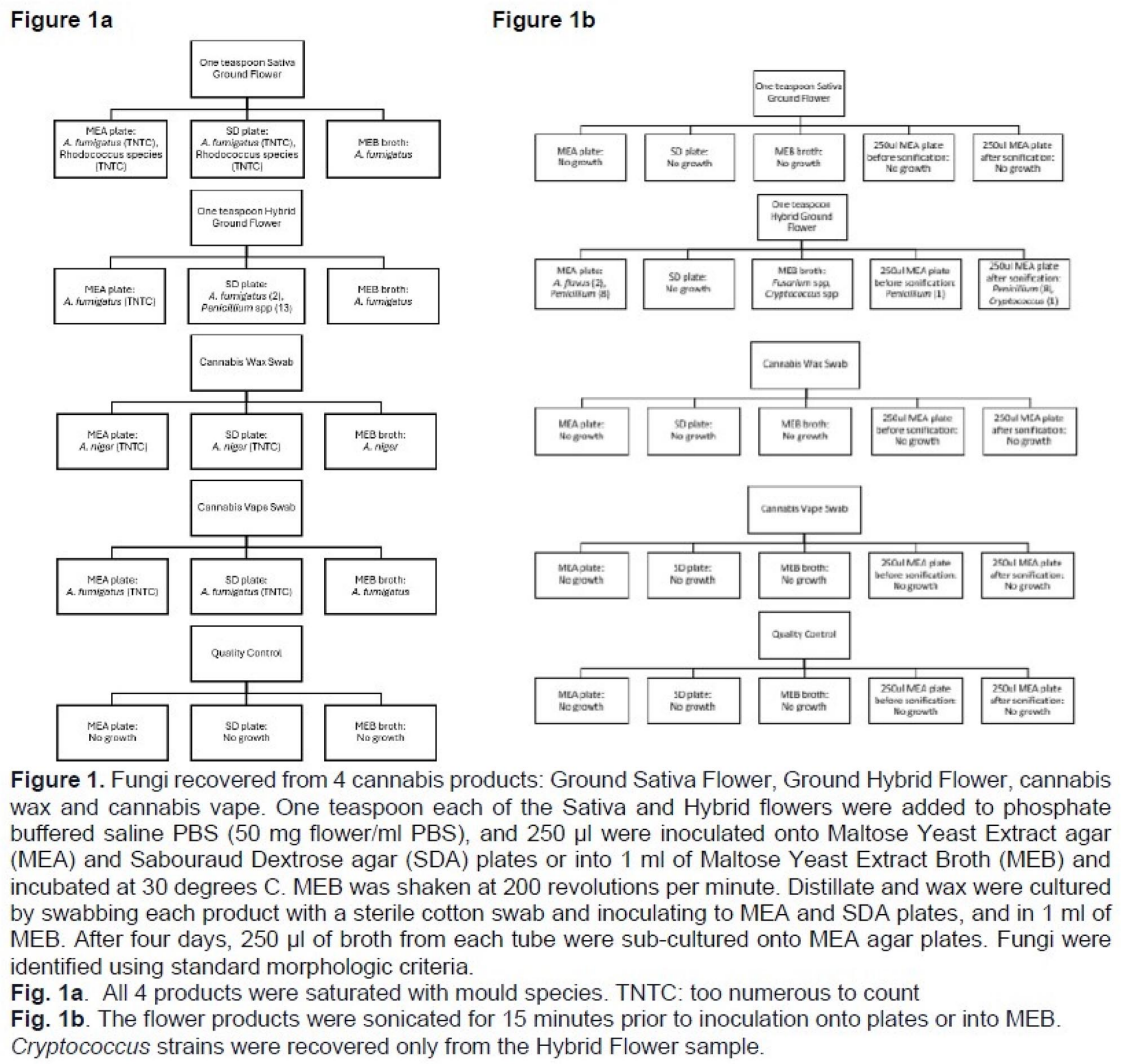

**Figure:**
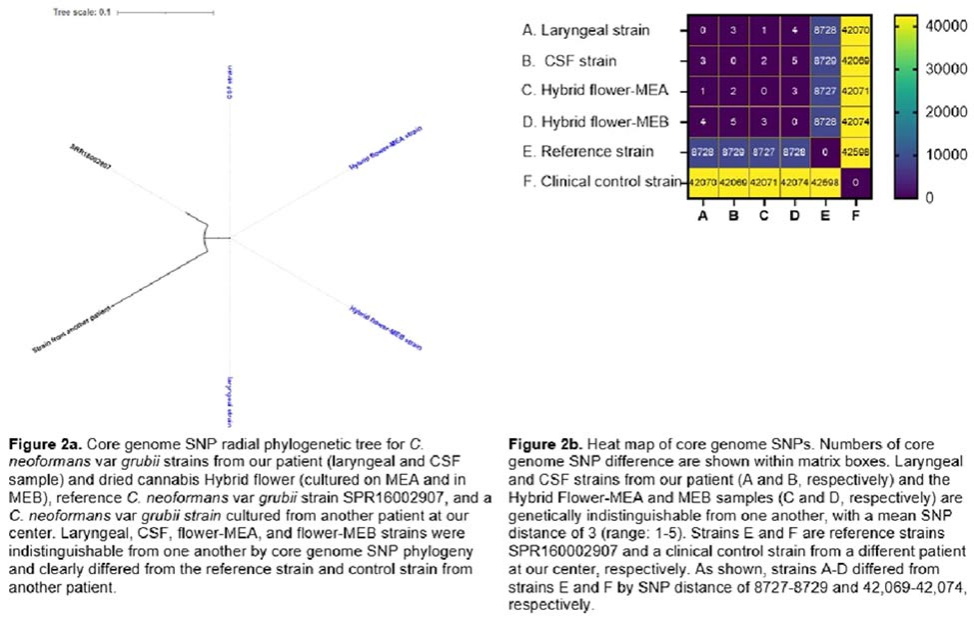

**Methods:**

Methods. We recovered *C. neoformans* var. *grubii* from cerebrospinal fluid (CSF) and larynx of a patient (pt) receiving chemotherapy for multiple myeloma. The pt provided dried ground cannabis flowers (Sativa and hybrid), cannabis vape (distillate) and cannabis wax that were purchased legally from different dispensaries. We cultured samples on maltose yeast extract agar (MEA) and Sabouraud plates or in maltose yeast extract broth (MEB). Strains underwent Illumina whole genome sequencing.

**Results:**

Results. Moulds were cultured readily from all 4 cannabis products (*Aspergillus fumigatus*, *A. flavus*, *A. niger, Fusarium* spp, *Penicillium* spp). After adding a sonication step and isolating mould colonies early to prevent overgrowth, we cultivated 2 *C. neoformans* var. *grubii* colonies from hybrid flower (but not other products) on MEA and in MEB. Strains from larynx, CSF, flower-MEA and flower-MEB were indistinguishable by core genome SNP phylogeny, but they differed significantly from a reference strain and a strain from another pt. Therefore, *C. neoformans* var. *grubii* from cannabis flower was the cause of cryptococcosis. Disseminated cryptococcois likely originated from inhalation of conidia and laryngeal infection. Crypotococcal conidia are larger than those of *Aspergillus* (4-6 µm, up to 100 µm with enlarged capsule vs. 2-3.5 µm), which may have prediposed to upper airway deposition. We are currently performing metagenomic sequencing of DNA from cannabis products to determine the full spectrum of fungal contamination.

**Conclusion:**

Conclusions. This is the first study to definitively link IFI or bacterial infection to a pathogen in medical cannabis used by the pt. Our data prove that cannabis consumption is a risk for IFI, as long postulated. There are no U.S. federal guidelines for development, medical use or quality testing of cannabis. Better regulation of medical cannabis is needed, as are rigorous clinical studies of efficacy and complications.

**Disclosures:**

Cornelius J. Clancy, MD, Merck: Grant/Research Support|Shionogi: Advisor/Consultant M Hong Nguyen, MD, Basilea: Advisor/Consultant|BioMerieux: Grant/Research Support|Melinta: Grant/Research Support|Pulmocide: Advisor/Consultant|Pulmocide: Grant/Research Support

